# Correlation of Lymph Node Characteristics and Extranodal Extension in Oral Cavity Squamous Cell Carcinoma

**DOI:** 10.1002/oto2.70032

**Published:** 2024-10-17

**Authors:** Piper A. Wenzel, Steven L. Van Meeteren, Nitin A. Pagedar, Marisa R. Buchakjian

**Affiliations:** ^1^ Department of Otolaryngology–Head and Neck Surgery University of Iowa Hospitals and Clinics Iowa City Iowa USA; ^2^ University of Iowa Holden Comprehensive Cancer Center Iowa City Iowa USA; ^3^ Present address: Department of Otolaryngology University of Michigan Ann Arbor Michigan USA

**Keywords:** extracapsular extension, extracapsular spread, extranodal extension, lymph node metastasis, oral cavity squamous cell carcinoma

## Abstract

**Objective:**

Identify correlations between lymph node characteristics and extranodal extension (ENE).

**Study Design:**

Retrospective chart review.

**Setting:**

Tertiary care center.

**Methods:**

Patients who underwent neck dissection for oral cavity squamous cell carcinoma from 2004 to 2018 were included, with a starting sample of 496. The primary outcome was ENE in at least 1 lymph node. Additional variables included number of dissected nodes, positive nodes by level, positive lymph node ratio (LNR), and diameter of metastatic deposit and ENE focus. Univariate and multivariate binary logistic regression analyses were performed to determine correlations between included variables and ENE.

**Results:**

Of the 496 patients, 233 had nodal metastasis (47.0%). 13,814 nodes were removed, with 714 (5.2%) containing metastasis. Of the positive nodes, 28.0% had ENE, 47.2% did not have ENE, and 24.8% were unknown. The mean ENE diameter was 5.1 mm (SD, 9.9). On univariate logistic regression analysis, ipsilateral neck LNR per 0.1 unit increase (odds ratio [OR] 1.16, 95% confidence interval [CI] 1.02‐1.32, *P* = .02), metastatic deposit size per 1 mm increase (OR 1.06, CI 1.04‐1.08, *P* < .0001), and clinical T‐ (*P* = .02) and N‐class (*P* = .0003) significantly correlated with ENE. On multivariate logistic regression analysis, size of metastatic deposit (OR 1.06, CI 1.03‐1.08, *P* < .0001) remained significantly correlated with ENE.

**Conclusion:**

Controlling for confounding variables, size of metastatic deposit was an independent predictor of ENE, suggesting that as the metastatic deposit size increases, the odds of extension through the capsule also increases. This may be due to capsule thinning as the deposit grows or could represent the invasive nature of aggressive disease.

Extranodal extension (ENE), defined as tumor extension beyond the lymph node capsule, is considered an important prognostic factor for recurrence and survival in oral cavity squamous cell carcinoma (OCSCC).^1^, ^2^, ^3^ The eighth edition of the American Joint Committee on Cancer (AJCC) staging manual has incorporated ENE as a poor prognostic indicator, reflecting the significance of ENE for cancer management.^4^ N‐classification is affected by the presence or absence of ENE but not by quantification of the size of ENE.

Not only is the presence of ENE important, but also the extent has been shown to affect prognostic outcomes. Specifically, ENE classified as major (>2 mm) has been associated with worse overall survival compared to minor (≤2 mm) ENE.^5^, ^6^ Previous reports have documented the mean size of ENE to be 2 mm (range, 1‐10) in head and neck SCC and 3.5 mm (SD 4.1) in OCSCC.^3^, ^5^ It has been suggested that future staging systems incorporate stratification by ENE extent.^5^

Lymph node ratio (LNR), defined as the ratio of lymph nodes positive for metastasis to total number of lymph nodes removed, has been reported to impact survival outcomes in OCSCC as well.^7^, ^8^, ^9^ Specifically, LNR has been demonstrated to be significantly associated with overall survival, progression‐free survival, disease‐free survival, and distant metastasis‐free survival.^8^, ^9^ High LNR and the presence of ENE have been associated in some studies while LNR was not found to be a predictor of ENE in Mair et al.^9^, ^10^, ^11^, ^12^ Additional studies are needed to further elucidate the relationship between LNR and ENE.^9^ Size of the metastatic deposit is another lymph node characteristic that has been reported to be correlated with the presence of ENE.^1^, ^2^, ^3^, ^13^, ^14^

Much of the currently available literature identifying relationships between lymph node characteristics and ENE is dated, has a small sample size, or focuses broadly on head and neck squamous cell carcinoma rather than OCSCC. This study aims to include a sizeable group of patients to provide updated information focused specifically on OCSCC treated with upfront curative‐intent surgery.

## Methods

### Study Population

A retrospective chart review was conducted following approval from the University of Iowa institutional review board. Adult patients who underwent upfront curative‐intent surgery for OCSCC at the University of Iowa Hospitals and Clinics between December 2004 and August 2018 were identified from the institutional tumor registry and eligible for inclusion. Clinical notes, radiology and pathology reports, and tumor registry data were reviewed.

Exclusion criteria included the absence of neck dissection, operation performed without curative intent or for recurrent disease, pathology demonstrating histology other than squamous cell carcinoma, primary tumor outside the oral cavity, patient with second upper aerodigestive tract cancer within five years, index cancer treated previously with radiation, cancer initially treated surgically at a different institution, or presence of gross disease remaining after surgery.

### Outcomes

The primary outcome was the presence of ENE in at least 1 lymph node, as determined from the patient's pathology report. ENE was defined as tumor extension beyond the lymph node capsule and into the surrounding connective tissue.^2^

Some pathology reports did not clearly document the presence or absence of ENE in each metastatic node. Nodes with unclear ENE status were recorded as “unknown.” For example, some pathology reports included information in the following format: “Lymph nodes, left neck levels 1A and 1B, resection: Metastatic squamous cell carcinoma in three of six lymph nodes (3/6). Largest metastasis 2.0 cm, with extranodal extension <0.1 cm from the capsule.” The 2 lymph nodes that were malignant but not described further would be considered to have “unknown” ENE status as the report did not clearly state if ENE was present.

Lymph node characteristics included neck levels dissected, total number of dissected nodes, positive nodes by level, positive LNR, diameter of metastatic deposit, number of lymph nodes with ENE, and diameter of ENE focus. In cases where reports did not separate the nodes by level, lymph nodes were recorded in the lowest numbered level noted. LNR was defined as the number of pathologically positive lymph nodes divided by the number of removed lymph nodes per ipsilateral neck.^7^ Size of ENE focus <1 and 1 mm were combined and quantified as 1 mm.

T‐ and N‐classifications for the seventh and eighth edition of the AJCC staging system were recorded from review of pathology reports, imaging reports, and clinical notes.^4^

### Statistical Analysis

SAS 9.4 was used to perform statistical analyses. Univariate and multivariate binary logistic regression analyses were used to identify correlations between lymph node variables and presence of ENE. *P* < .05 was considered statistically significant.

## Results

### Study Population

The initial population size was 496 OCSCC patients from which 13,814 lymph nodes were dissected. Of the total dissected nodes, 714 (5.2%) were positive for metastasis. The 714 positive nodes were from a subset of 233 patients (47.0%) in which 154 (66.1%) were male with a mean age of 60.5 years (SD, 12.5; range, 29‐94). Among the 233 patients with at least 1 metastatic node, 122 (52.4%) had ENE.

In the subset of 233 patients with at least 1 positive node, the most common primary tumor sites were oral tongue (48.5%), floor of mouth (20.5%), and alveolus (14.9%). The histologic grade was determined to be moderate in most patients (62.2%). Lymphovascular invasion (LVI) was identified in 50.7% of patients and perineural invasion (PNI) in 57.0%. Bone invasion was present in 32.5% of patients. Tumor depth of invasion was >5 to 10 mm in 33.3% of patients and >10 mm in 44.4% of patients with at least 1 positive node.

The most common clinical T‐classifications (AJCC eighth edition) were T4a (41.2%) and T2 (26.2%). The most common clinical N‐classifications (AJCC eighth edition) were N0 (33.9%), N1 (20.2%), and N2b (20.2%). Pathologically, the most common T‐ and N‐classifications were T4a (50.6%) and N3b (52.4%). Detailed T‐ and N‐classification data is provided in the supplemental materials.

The remainder of the analysis focused on the positive lymph nodes from the subset of 233 OCSCC patients with nodal metastasis.

### Lymph Node Characteristics

The distribution of positive nodes by level was level 1A and 1B 34.6%, level 2A and 2B 37.5%, and levels 3 to 6 27.9% (Table 1). The mean number of nodes in the respective level of a metastatic node was 8.0 (SD, 5.4; range, 1.0‐35.0). The mean number of malignant nodes in the respective level of a metastatic node was 2.3 (SD, 1.8; range, 1.0‐13.0). The size of lymph node metastasis was available for 399 nodes, and the mean diameter of metastatic focus was 13.1 mm (SD, 12.1; range, 1.0‐71.0) (Table 2).

**Table 1:** Metastatic Lymph Nodes by Level

Lymph node level	Frequency	Percent	Cumulative frequency	Cumulative percent
1A and 1B	247	34.6	247	34.6
2A and 2B	268	37.5	515	72.1
3	148	20.7	663	92.9
4	47	6.6	710	99.4
5	3	0.4	713	99.9
6	1	0.1	714	100.0

**Table 2:** Descriptive Statistics of Metastatic Nodes

Variables	N	Mean	SD	Minimum	Maximum
Positive LNR on ipsilateral side	714	0.2	0.2	0.02	1.00
Number of nodes removed from ipsilateral side	714	27.5	12.0	1.0	66.0
Number of nodes in respective level	714	8.0	5.4	1.0	35.0
Number of malignant nodes in respective level	714	2.3	1.8	1.0	13.0
Diameter of metastatic deposit (mm)	399	13.1	12.1	1.0	71.0

Abbreviations: LNR, lymph node ratio; SD, standard deviation.

### Characteristics of Lymph Nodes With ENE

Among the 714 metastatic nodes, 200 (28.0%) were documented to have ENE in at least 1 node, 337 (47.2%) did not have ENE, and 177 (24.8%) were unknown. Of the metastatic nodes, 484 (67.8%) were from a patient with recorded ENE in at least 1 lymph node. The mean ENE size, available for 38 nodes, was 5.1 mm (SD, 9.9) with a minimum and maximum of 1 and 48 mm, respectively. Additional analyses on ENE size could not be performed due to data only being available for 38 nodes.

Of note, there were 36 nodes with ENE in 27 patients clinically staged N0. One patient had 4 such nodes, 1 patient had 3 nodes, and 4 patients had 2 nodes. The majority of these patients had a primary tumor of the oral tongue (55.6%). LVI and PNI were present in 51.9% and 63.0% of these patients, respectively. The most common clinical T‐classifications in these patients were T1 (37.0%) and T4a (33.3%).

### Univariate Analysis of Metastatic Node Characteristics With Presence of ENE

Univariate logistic regression analyses were performed for metastatic nodes in which ENE was clearly documented in the pathology report as present or absent (n = 537, 75.2%). The nodes (n = 177) for which ENE status was unknown were excluded from the analyses.

On univariate analysis, LNR in the ipsilateral neck per 0.1 unit increase (odds ratio [OR] 1.16, confidence interval [CI] 1.02‐1.32, *P* = .024) was significantly correlated with the presence of ENE in metastatic nodes (Table 3). In other terms, with every 0.1 unit increase in LNR, the odds of ENE in that node increased by 1.16. Size of metastatic deposit per 1 mm increase (OR 1.06, CI 1.04‐1.08, *P *< .0001) (n = 382) was also significantly correlated with ENE presence. With each 1 mm increase in size of metastatic deposit, the odds of ENE increased by 1.06. For example, a node with a 15 mm deposit would have 1.34 times the odds of ENE compared to a node with a 10 mm deposit.

**Table 3:** Univariate Analysis of Metastatic Node Characteristics Correlated With Presence of ENE

Variable		OR	95% CI	*P* value
Node in levels 3‐6	Compared to levels 1‐2	0.84	0.56‐1.26	.40
LNR in ipsilateral neck per 0.1 unit increase		1.16	1.02‐1.32	.024
Number of lymph nodes on ipsilateral side		1.00	0.98‐1.01	.57
Number of lymph nodes in respective level		0.99	0.96‐1.03	.71
Number of malignant lymph nodes in respective level		1.06	0.93‐1.21	.37
Size of metastatic deposit per 1 mm increase	n = 382	1.06	1.04‐1.08	**<.0001**
Clinical T class (eighth edition)				**.015**
T2	Compared to T1	0.70	0.37‐1.30	
T3	Compared to T1	0.67	0.34‐1.33	
T4a	Compared to T1	1.26	0.73‐2.20	
T4b	Compared to T1	3.36	0.77‐14.63	
Clinical N class (eighth edition)				**.0003**
N1	Compared to N0	1.38	0.76‐2.50	
N2b	Compared to N0	1.71	1.01‐2.90	
N2c	Compared to N0	2.22	1.33‐3.72	
N3b	Compared to N0	4.21	2.18‐8.14	

Analyses were performed for 537 nodes for which ENE status is known. Size of metastatic deposit analysis was performed for 382 nodes for which ENE status and metastatic deposit size was known. Bold text indicates *P* ≤ .05.

Abbreviations: CI, confidence interval; ENE, extranodal extension; LNR, lymph node ratio; OR, odds ratio.

Clinical T‐classes (AJCC eighth edition) T2, T3, T4a, and T4b compared to T1 were correlated with ENE presence (*P* = .015). ORs (95% CI) were 0.70 (0.37‐1.30) for T2, 0.67 (0.34‐1.33) for T3, 1.26 (0.73‐2.20) for T4a, and 3.36 (0.77‐14.63) for T4b. Clinical N‐classes (AJCC eighth edition) N1, N2b, N2c, and N3b compared to N0 were correlated with ENE presence (*P* = .0003). ORs (95% CI) were 1.38 (0.76‐2.50) for N1, 1.71 (1.01‐2.90) for N2b, 2.22 (1.33‐3.72) for N2c, and 4.21 (2.18‐8.14) for N3b.

Variables that were not predictors of ENE included the presence of metastatic node in levels 3‐6 compared to levels 1 to 2, number of malignant lymph nodes in a respective level, and total number of lymph nodes dissected on the ipsilateral side of the neck and in the respective level.

### Multivariate Analysis of Metastatic Node Characteristics With Presence of ENE

Multivariate logistic regression analyses were performed for metastatic nodes in which metastatic deposit size and ENE status was available (n = 382). On multivariate analysis, size of metastatic deposit per 1 mm increase (OR 1.06, CI 1.03‐1.08, *P* < .0001) remained predictive of the presence of ENE in metastatic nodes when controlling for other important characteristics such as LNR in the ipsilateral side, node level, and clinical T‐ and N‐class (Table 4). LNR in the ipsilateral neck per 0.1 unit increase and clinical T‐ and N‐class (AJCC eighth edition) were not independent predictors of ENE presence.

**Table 4:** Multivariate Analysis of Metastatic Node Characteristics Associated With Presence of ENE

Variable		OR	95% CI	*P* value
LNR in ipsilateral neck per 0.1 unit increase		1.19	0.99‐1.43	.058
Size of metastatic deposit per 1 mm increase		1.06	1.03‐1.08	<.0001
Clinical T class (eighth edition)				.31
T2	Compared to T1	0.72	0.34‐1.49	
T3	Compared to T1	0.53	0.23‐1.22	
T4a	Compared to T1	0.88	0.44‐1.77	
T4b	Compared to T1	2.38	0.37‐15.52	
Clinical N class (eighth edition)				.18
N1	Compared to N0	1.05	0.54‐2.05	
N2b	Compared to N0	1.20	0.57‐2.50	
N2c	Compared to N0	1.24	0.62‐2.48	
N3b	Compared to N0	2.87	1.20‐6.86	

Analyses were performed for 382 metastatic nodes for which metastatic deposit size and ENE status are known. Bold text indicates *P* ≤ .05.

Abbreviations: CI, confidence interval; ENE, extranodal extension; LNR, lymph node ratio; OR, odds ratio.

## Discussion

This study is a large retrospective analysis of surgically treated OCSCC focusing on the role of lymph node characteristics in predicting the presence of ENE in 714 metastatic nodes. Our results indicated that the size of metastatic deposit per 1 mm increase was an independent risk factor for the presence of ENE in metastatic nodes. Variables that were not independently correlated with the presence of ENE included LNR in the ipsilateral neck, clinical T‐ and N‐classifications, node level, number of lymph nodes on the ipsilateral side and in the respective level, and number of metastatic nodes in the respective level.

Regarding the correlation between an increase in metastatic deposit size and the presence of ENE, our findings are consistent with but also extend from those previously reported in historical studies.^1^, ^13^, ^14^ A study evaluating 431 nodes positive for head and neck SCC found an association between larger lymph node diameter and incidence of ENE (*P* < .001).^3^ The median diameter of lymph nodes was 9 mm (range, 1‐27 mm), smaller but similar to our results of a mean metastatic focus diameter of 13.1 mm. Another study published 11 years later evaluated 212 OCSCC patients with nodal metastasis and found a significant positive correlation between size of metastatic deposit in the node and presence of ENE (*r*_s_ = .551, *P* < .001).^2^ To our knowledge, our study is the first to show an increase in risk of ENE with each continuous 1 mm increase in size of metastatic deposit.

Interestingly, in our subset of 233 patients with nodal metastasis, there were 36 nodes with ENE in 27 patients who were clinically staged N0. These findings are similar to those of Mair et al. who found the incidence of nodal metastasis to be 28.5% (101/354) in 354 clinically node negative oral cancer patients and presence of ENE in 15.3% of the 354 patients.^12^ We concur with their conclusion of the importance of elective neck dissections to accurately stage clinically N0 patients. Of note, the study additionally found metastatic node size >15 mm to be a predictor of ENE (*P* = .018) while LNR was not predictive, both results in agreement with our study.

The correlation we demonstrate between lymph node metastatic deposit size and ENE may provide benefits for patient counseling and operative and treatment planning. There are no imaging modalities that can detect ENE with complete accuracy, but patients with evidence of large lymph nodes preoperatively may be counseled that they have an increased odds of ENE presence, contributing to a worse prognosis.^15^ The improved prediction of risk may provide patients with a more complete understanding and expectation for their treatment course prior to undergoing surgery for definitive pathologic assessment.

The current standard of care in patients with evidence of pathological ENE is to undergo cisplatin chemotherapy, which confers additional risk and requires pretreatment medical evaluation.^4^, ^16^ The recommendations from the National Comprehensive Cancer Network are that postoperative adjuvant therapy should begin within 6 weeks after surgery.^17^ Delays in initiation of adjuvant therapy beyond 6 weeks have been associated with worse overall survival, recurrence‐free survival, and locoregional control.^18^ As surgical pathology can often take several days to be reported, the ability to predict ENE prior to surgery may help to inform referrals of patients with elevated risk of ENE to medical oncologists, preventing delays in initiation to adjuvant therapy.

Limitations of this study include the retrospective design with the involvement of data from a single tertiary care center. Additionally, data collection was limited by the information documented in pathology reports and the electronic medical record. As described in the methods section, ENE status and size of metastatic deposit and ENE were not explicitly stated for each lymph node removed. The small number of nodes (38) with ENE size available limited our ability to perform additional relevant analyses. Additionally, pathology reports sometimes group nodes into multiple levels rather than detailing the number of nodes from each specific level.

## Conclusions

This retrospective analysis provides insight into important clinicopathologic details of lymph node metastases in OCSCC. Controlling for potential confounding variables, size of metastatic deposit was an independent predictor of ENE presence. This information may be beneficial for adjuvant treatment planning and could play a role in counseling patients as to the likelihood of recommending concurrent chemoradiation after surgery.

## Author Contributions

**Piper A. Wenzel**, methodology, investigation, writing—original draft; **Steven L. Van Meeteren**, investigation, writing—review and editing; **Nitin A. Pagedar**, data analysis, writing—review and editing; **Marisa R. Buchakjian**, conceptualization, methodology, investigation, writing—review and editing, and supervision.

## Disclosures

### Competing interests

None.

### Funding source

None.

## Supporting information

Supporting information.
