# Supplementary material for: Correlation of Lymph Node Characteristics and Extranodal Extension in Oral Cavity Squamous Cell Carcinoma
Source: OTO Open. 2024 Oct 17;8(4):e70032. doi: 10.1002/oto2.70032 (PMC11487070; doi:10.1002/oto2.70032)
Supplement: Supplementary file 1 — Supporting information. [file OTO2-8-e70032-s001.docx]

**SUPPLEMENTAL**

| Table 1. Clinical and pathologic staging | | |
| --- | --- | --- |
| Variable | **Frequency** | **Percent** |
| T class (7^th^ edition) |  |  |
| T1 | 44 | 18.9 |
| T2 | 60 | 25.8 |
| T3 | 30 | 12.9 |
| T4a | 94 | 40.3 |
| T4b | 5 | 2.2 |
| N class (7^th^ edition) |  |  |
| N0 | 78 | 33.5 |
| N1 | 52 | 22.3 |
| N2a | 5 | 2.2 |
| N2b | 60 | 25.8 |
| N2c | 37 | 15.9 |
| N3 | 1 | 0.4 |
| Clinical T class (8^th^ edition) |  |  |
| T1 | 41 | 17.6 |
| T2 | 61 | 26.2 |
| T3 | 30 | 12.9 |
| T4a | 96 | 41.2 |
| T4b | 5 | 2.2 |
| Clinical N class (8^th^ edition) |  |  |
| N0 | 79 | 33.9 |
| N1 | 47 | 20.2 |
| N2a | 5 | 2.2 |
| N2b | 47 | 20.2 |
| N2c | 33 | 14.2 |
| N3b | 22 | 9.4 |
| Pathologic T class (8^th^ edition) |  |  |
| T1 | 28 | 12.0 |
| T2 | 55 | 23.6 |
| T3 | 30 | 12.9 |
| T4a | 118 | 50.6 |
| T4b | 2 | 0.9 |
| Pathologic N class (8^th^ edition) |  |  |
| N1 | 50 | 21.5 |
| N2a | 3 | 1.3 |
| N2b | 47 | 20.2 |
| N2c | 11 | 4.7 |
| N3a | 0 | 0 |
| N3b | 122 | 52.4 |
